# Regulated deficit irrigation: an effective way to solve the shortage of agricultural water for horticulture

**DOI:** 10.1007/s44154-022-00050-5

**Published:** 2022-07-25

**Authors:** Bohan Yang, Peining Fu, Jiang Lu, Fengwang Ma, Xiangyu Sun, Yulin Fang

**Affiliations:** 1 College of Enology, Shaanxi Provincial Key Laboratory of Viti-Viniculture, Viti-viniculture Engineering Technology Center of State Forestry and Grassland Administration, Shaanxi Engineering Research Center for Viti-Viniculture, Heyang Viti-viniculture Station, Ningxia Eastern Foot of Helan Mountain Wine Station, Northwest A&F University, Yangling, 712100 China; 2 Center for Viticulture and Enology, School of Agriculture and Biology, Shanghai Jiao Tong University, Shanghai, 200240 China; 3 State Key Laboratory of Crop Stress Biology for Arid Areas, Shaanxi Key Laboratory of Apple, College of Horticulture, Northwest A&F University, Yangling, 712100 China

**Keywords:** Regulated deficit irrigation, Horticulture, Water use efficiency, Crop quality

## Abstract

The deficient agricultural water caused by water shortage is a crucial limiting factor of horticultural production. Among many agricultural water-saving technologies, regulated deficit irrigation (RDI) has been proven to be one of the effective technologies to improve water use efficiency and reduce water waste on the premise of maintaining the quality of agricultural products. RDI was first reported more than 40 years ago, although it has been applied in some areas, little is known about understanding of the implementation method, scope of application and detailed mechanism of RDI, resulting in the failure to achieve the effect that RDI should have. This review refers to the research on RDI in different crops published in recent years, summarizes the definition, equipment condition, function, theory illumination, plant response and application in different crops of RDI, and looks forward to its prospect. We expect that this review will provide valuable guidance for researchers and producers concerned, and support the promotion of RDI in more horticultural crops.

## Introduction

The shortage of water resources had become the consensus of all mankind (Deng et al. 2006; Schiermeier 2014). Agricultural water consumption was the most important freshwater consumption of human (Ewaid et al. 2020; Singh et al. 2019), accounting for more than 2/3 of the total human freshwater consumption every year (Chai et al. 2016). The shortage of water resources would inevitably lead to the shortage of agricultural water, and the waste caused by unscientific agricultural water use would aggravate the shortage of water resource (Chai et al. 2014). Therefore, how to improve the effective utilization rate of agricultural water and rationalize the use of water resources in agricultural production was of great significance for human beings, especially for regions in arid and semi-arid areas (Forouzani and Karami 2011; Li and Qian 2018).

Among many agricultural water-saving technologies, regulated deficit irrigation (RDI) had been proved to be one of the effective technologies to improve water use efficiency and reduce water waste on the premise of maintaining the quality of agricultural products, especially in the horticultural crops represented by fruits and vegetables (Edwards and Clingeleffer 2013; Wakrim et al. 2005). Hence, this review summarized that RDI as a water-saving irrigation method from the basic situation of RDI, the specific function of RDI in production, the principle of action and the response off plants to it, as well as the application of RDI in horticultural crops, and promotes the promotion of RDI in production.

## Definition and main approaches of RDI

RDI was first appeared by Chalmers et al. (1981), and then Australian researchers firstly used and defined RDI to describe related work as a term in 1984 (Mitchell et al. 1984). There were differences in the definition and effect of RDI among different researchers. However, the basic definition of RDI was generally accepted, that is, in the whole growth period, or in a specific phenological period of plants, irrigation was less than the optimal amount of irrigation needed for plant vegetative growth. The amount of irrigation was usually set based on the percentage of estimated crop evapotranspiration (ETc) (Allan et al. 1998). It is generally accepted that the total amount of irrigation set for RDI should be no higher than 70% ETc, but there is no clear consensus on the amount of irrigation at different periods. This irrigation method could effectively reduce the total amount of irrigation water. And furthermore, some studies have shown that RDI could make plants in a way that was more inclined to reproductive growth to deal with appropriate water deficit, which was more conducive to people’s needs. So it could improve crop quality (Romero and Martinez-Cutillas 2012), finally achieve the purpose of improving orchard economic benefits (Lima et al. 2019; Terry and Kurtural 2011).

In recent years, the related research on RDI had been gradually increased and enriched, and it had been tested or applied in a variety of crops. Such as citrus (Gonzalez-Dugo et al. 2018; Romero-Trigueros et al. 2017, 2020), grape (da Silva et al. 2018; Guo et al. 2021; Ju et al. 2019), blueberry (Keen and Slavich 2012; Lobos et al. 2016), pear (Lepaja et al. 2018; Wu et al. 2020), mango (Dos Santos et al. 2016, 2020), peach (Falagan et al. 2016; Manuel Miras-Avalos et al. 2016, 2017), melon (Lamaoui et al. 2018; Zeinalipour et al. 2017) and other fruits, as well as tomato (Bogale et al. 2016; Coyago-Cruz et al. 2019; Hooshmand et al. 2019; Wang and Zhang 2014), lettuce (Chang et al. 2021; Lin et al. 2020), sugar beet (Abyaneh et al. 2017; Li et al. 2019), cauliflower (Abdelkhalik et al. 2019a; Hachmann et al. 2019), pepper (Abdelkhalik et al. 2020; Yang et al. 2018), potato (Xie et al. 2012) and other vegetables. Some food crops such as wheat (El-Sanatawy and Zedan 2020; Ma et al. 2019; Zhang et al. 2021), maize (Ma et al. 2016; Salem et al. 2021) and horticultural flowers such as marigold flower (Yasheshwar et al. 2017) also had related studies on the application of RDI. With the deepening of the study, the physiological basis of the effects of RDI on plants and the response of plants had become increasingly clear.

In a broad sense, there were two kinds of common RDI methods: Stage-based deficit irrigation, and Partial root-zone irrigation/Partial root drying (PRD). In recent years, the Subsurface irrigation method could also effectively achieve the purpose of water-saving irrigation, hence it was included in the scope of RDI in this review. In a narrow sense, RDI generally refered to Stage-based deficit irrigation, which was distinguished from the other two methods.

### Stage-based deficit irrigation

**Fig. 1:**
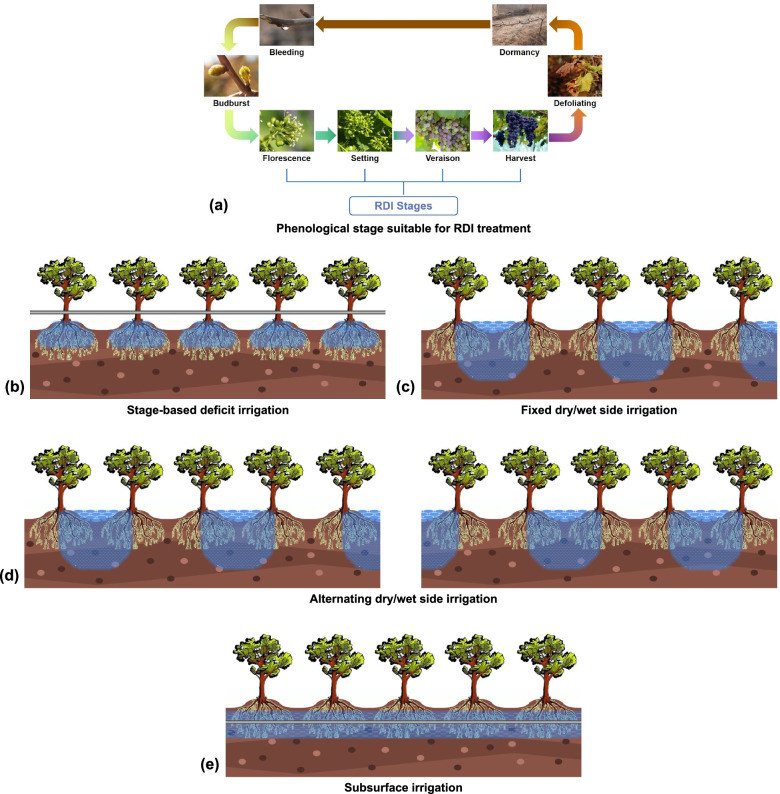
Three kinds of RDI methods. **a** Phenological stage suitable for RDI treatment; **b** Stage-based deficit irrigation; **c** Fixed dry/wet side irrigation; **d** Alternating dry/wet side irrigation; **e** Subsurface irrigation

This kind of irrigation methods appeared earliest and the easiest. According to the different water requirements of different phenological periods, the basic way was to carry out water deficit irrigation in the set critical period, so that it could achieve the purpose of water saving to a certain extent without affecting the basic growth of plants. The basic principle of this method was that the water demand of plants and the effects of water deficit on plants at different growth stages were different. In the non-critical stages, the amount of irrigation water of plants was less than that required, which could not only avoid ineffective and excessive vegetative growth, but also promoted reproductive growth under mild stress, by the functional secondary metabolites such as hormones and polyphenols (Fig. 1a, b). Previous studies had shown that RDI in a specific stages could improve fruit quality while saving water moderately. RDI of wine grapes during the stage from veraison to maturity could significantly increase the contents of anthocyanins and phenols in berries (Ju et al. 2019). These substances were critical for the quality of wine grapes and were beneficial to human health (Liang et al. 2008; Oliveira et al. 2019; Sun et al. 2020). However, some studies had shown that RDI could reduce fruit yield (or single berry weight) (Lu et al. 2019). Therefore, it was necessary to assess in advance what the key economic indicators of the crop include, and not all plants were suitable for this RDI method.

According to the previous research, compared with different plants, different phenological stages of the same plant and different degrees of deficit in the same phenological stages, the response of plants to water deficit were different. Therefore, the key point of this RDI method was how to determine the water requirement of the target plant in different phenological stages and the effect of water shortage. For example, wheat at tillering, stem elongation, and grainfilling growth stages was more sensitive to water deficit than at dormant stage (Wang et al. 2005), and wheat responded to water deficit more sensitively in post-tillering than in the early stage (Kang et al. 2002). If the stage of RDI was not selected properly, the growth of wheat would be limited and the yield would be greatly reduced. Another example, if the grape was short of water during the berry expansion stage, the yield would also be reduced (McCarthy 2000). Although grape was one of the crops commonly used in RDI, it was mainly concentrated used in the veraison and after, which could save water and improve fruit quality without reducing the yield as little as possible.

### Partial root-zone irrigation/Partial root drying (PRD)

PRD made plants in the state of water deficit, so it was also a kind of RDI in a broad sense. Its basic way was different from that of Stage-based deficit irrigation in which the whole root system was irrigated according to the period, but only half of the root system is irrigated at one time while the other half root system is in a state of drought. In the operation, it is generally divided into fixed dry/wet side irrigation (Fig. 1c) and alternating dry/wet wide irrigation (Fig. 1d).

The basic principle of this irrigation method was to make the plant root system in a state of uneven growth. The drought part of the root system would respond to drought stress, produce and transport stress-related hormones to plant leaves, coordinated stomatal closure and reduce transpiration. On the other hand, the wet part of the root system could ensure that the plant get enough water to meet the basic growth needs of the plant. Therefore, for the whole plant, this method could reduce the water demand of plants by causing plant stress resistance response and reducing transpiration, so the corresponding irrigation amount could be reduced to achieve the purpose of water-saving irrigation (Liu et al. 2006b; Sobeih et al. 2004). However, its specific effect, application scope and operation mode in different plants also need to be further studied by future researchers.

### Subsurface irrigation

Subsurface irrigation was a new type method in recent years (Fig. 1e). Most of the traditional water-saving irrigation was carried out by laying drip irrigation pipes on the ground, while subsurface irrigation directly irrigates the root system by laying the pipes underground. The traditional RDI method was to infiltrate the soil around the underground roots from the surface, and then plant roots absorbed water from the soil. These methods had a low construction cost, but there would still be some ineffective irrigation to a certain extent. Subsurface irrigation ensure that water was absorbed and utilized by plants to the maximum extent, but the disadvantage was the high construction cost.

Its fundamental principle and effect on plants were different from the traditional RDI methods. Subsurface irrigation could induce plant hardening, cause mild water stress response, and then lead to plant morphological strengthening, epidermis thickening and producing more waxy layer. On tomato, compared with the traditional RDI methods, subsurface irrigation could not only improve the fruit quality, but also enhanced the photosynthetic activity and appropriately increase the fruit yield (Xu et al. 2011b). Summarily, this irrigation method could be used for field production in some areas to cope with low soil temperature in early spring and induce plants to improve yield and quality while saving water (Gan et al. 2013).

## Equipment and operation of RDI

### Irrigation system

The irrigation system of RDI should be set according to the type of RDI, crop type, requirement of water saving, and cultivation condition, and there is no restrict rules.

Irrigation system consists of head hub, water distribution network, irrigation device, electrical and control equipment (Table 1). The network may include main pipe and branch pipe, as well as a variety of control and regulation valves and safety devices. The system layout should be determined by analyzing position of water source and plantation, topography, geology, plants, buildings and other factors. The head hub should be located near the water source to facilitate water intake. Pipeline layout and installation should be as smooth as possible, less through obstacles and buildings. Reasonable flow calculation be carried out throughout the piping system to ensure that peak flow requirements are met. Branch pipe be arranged according to the direction of planting row, water meter be installed according to situation, easy to record the flow of each branch pipe. When drip irrigation is adopted, the drip holes should be set according to the spacing of plants, and ensure the uniform distribution of holes and uniformity of diameter. When flood irrigation is adopted, the irrigation amount of the whole area should be evenly.

**Table 1:** Equipment in RDI system

Irrigation system	Equipment	Note
Head hub	Pump	Near the water source
	Booster	
	Filter	
	Reservoir	
Water distribution network	Main pipe	Reasonable distribution; Meeting flow requirement
	Branch pipe	
	Connector	
Irrigation device/Drip hole	Different devices/Aperture depending on irrigation method	Evenly distribution; Same aperture
Electrical and control equipment	Master controller	Reasonable power and capability
	Regulating valve	
	Check valve	
	Drain valve	
	Safety device	

### Operation mode

Although the amount of irrigation was usually set based on the percentage of ETc, when planting without rain shelter, the amount of rainfall should be subtracted from the irrigation amount.

The operation mode of system should be carried out by rotation irrigation. The whole irrigation division is divided into small areas or units, and the system is divided into several irrigation groups, each group contains one or several basic units. The combination form mode of each group should be easy operation, reduce the design flow of transmission and distribution pipeline as far as possible, and make the maximum flow supply flow supply of the system not exceed the design, keep the condition of pump stable. Furthermore, the determination of the rotation irrigation sequence should take into account factors such as the distribution of plants and the convenient.

## The function of RDI in application

### Improving the utilization of water and nutrient resources

**Fig. 2:**
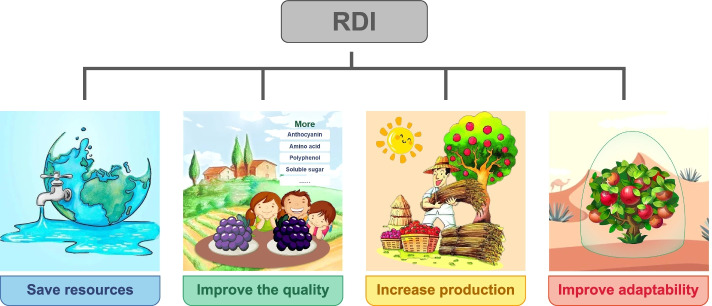
The four main functions of RDI in application

The function of RDI in application was shown as in Fig. 2. The unit of water use efficiency (WUE) was typically expressed in kilogram per hectare per millimeter. In some cases, the WUE was used to describe the efficiency of amount of irrigation applied to the crop (Li and Long 2019; Sun et al. 2010; Xu et al. 2021). Studies had shown that RDI could effectively improve plant water and nutrient utilization (Li et al. 2007; Wakrim et al. 2005; Wang et al. 2013). Compared with the normal irrigation group as a control, the water transpiration of the common beans in the RDI group was reduced, the lost by land transpiration was also reduced, and the WUE increased (Wakrim et al. 2005). The research on maize and tomato RDI treatments had confirmed that RDI could increase the ratio of nitrogen absorbed by plant to the nitrogen supplied, and improve the use efficiency (Li et al. 2007; Wang et al. 2013). Further studies had showed that this increasing was mightly due to the reduced soil bulk density and the percentage of water holding pores in the soil by RDI (El Baroudy et al. 2014), which promotes the absorption of nitrogen by plants. At the same time, RDI would also change the distribution of nitrogen, resulting in more nitrogen being distributed in the middle and upper part of the leaf canopy (Wang et al. 2010).

### Improving the economic quality of crops

At present, there were abundant researches on RDI to improve crop quality, especially for cash crops such as grapes. Due to the particularity of some cash crops, their quality and flavor had a greater impact on their economic value than yield. 50–70% ETc irrigation in wine grapes significantly increased monomeric anthocyanin contents by increasing the relative expression levels of key anthocyanin synthesis genes in the phenylalanine pathway (such as *VvF3H*, *VvUFGT*), but it did not reduce yield (Yang et al. 2020a), and it also increase the content of amino acid and their volatile compounds, fatty acids, and polyphenols (Ju et al. 2018a, b). These quality indicators played an important role in the quality of the processed wine grapes.

### Maintaining or increasing yield

The plants could return to normal growth and development levels in a short period of time when restored the normal irrigation after moderate RDI treatment, therefore, the impact on yield was minimal (Kang et al. 2000). And due to the existence of the compensation effect, it might be possible to increase the production. In the maize RDI experiment conducted by Dominguez et al., the treatment group (RDI in the vegetative growth device) compared with the control group (RDI in the whole period), the yield increased by 10–20% (Dominguez et al. 2012). Alternate RDI experiments on cotton confirmed that partial deficit irrigation could increase cotton yield by about 13–24% (Du et al. 2006).

### Improving plant adaptability

The period of RDI treatment and opportunity to resume normal irrigation had significant impact on the growth and development of plants. Therefore, compared with the fully irrigated group, the maize of the treatment group that was treated with water deficit for a period of time at the seedling stage and recovered afterwards had better adaptability to soil water deficit. But long-term severe water shortage would have a significant negative impact on plants (Siddique and Bramley 2014). However, applying a slight RDI treatment at the early stage of the growth could enhance the drought resistance of plants in the later stage and maintain drought resistance (Liu et al. 2006a).

## The mechanism of RDI theory

**Fig. 3:**
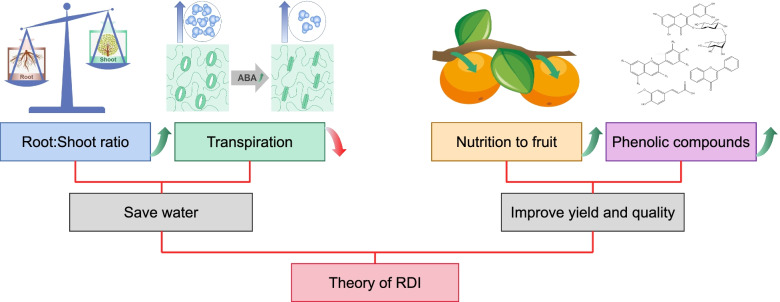
Mechanism of two functions of RDI theory

Regarding the action principle of RDI, there were many explanations with different focuses. Based on different purposes and results (saving water and improve yield and quality), this review roughly divided them into two types (Fig. 3).

### Theory of RDI to saving water

RDI could save irrigation water was an obvious phenomenon. The mainly theories about water saving in RDI were Root-Shoot (R/S) balance, which was believed that the root and shoot of plants were in a mutually dependent and competitive relationship, and the development of root and shoot was in a dynamic balance (Rogers et al. 1995; Thornley 1971), and the biomass partitioning was one of the mechanisms which plants deal with a resource-constrained environment (Bloom et al. 1985; Bonifas and Lindquist 2006). When the environmental conditions were abundant and stable, the ratio of root to shoot was in a relatively stable interval, and this value was mainly determined by the genetic factors of the plants (Wilson 1988). However, when the external nutritional conditions change or under stress, the plant would automatically adjusted the distribution of nutrients to minimize the damage to the environment and was most conducive to the continuation of its species (Bonifas et al. 2005). Therefore, in the stress environment, the root and shoot were in competition relationship (Agathokleous et al. 2019).

Hormones were a kind of microscale organic compounds that could regulate their own physiological processes, and thus played an crucial role in regulating the growth and development of plants (Davies et al. 1997; Ferrero et al. 2018; Gonzalez-Villagra et al. 2019). When the root was in a state of water deficit, under the regulation of some endogenous hormones (such as ABA), the aboveground part of the plant closed the stomata, decreased leaf area growth, reduced the shoot development to reduce water evaporation, which leaded to decrease of water demand (Alves and Setter 2000; Davies et al. 2002; Guo et al. 2020). At the same time, more assimilation products were transferred to the underground part, and the roots grew thicker, which improved the soil WUE of plant (Hooker and Thorpe 1997; Li et al. 2011). In recent years, the changes and effects of a variety of plant endogenous hormones during berry development had been widely studied (Deluc et al. 2007; Wheeler et al. 2009). Previous studies had shown that the content of ABA in wine grapes were increased during the veraison under RDI (Yang et al. 2020b), which had also been proved in other plants.

In summary, the basic principle of water-saving during RDI was the treatment influenced the root-shoot ratio of plants through the management of soil moisture, controlled the root growth and aboveground parts of plants at the same time, increased the utilization of plants, reduced the vegetative growth and transpiration, so as to reduce the water demand of plants and irrigation water.

### Theory of RDI to improve yield and quality

Water deficit affected plant growth by reducing carbon accumulation, cell number and tissue expansion (Tardieu et al. 2011). Most of research results have confirmed that the vegetative growth of fruit trees was greatly affected by water deficit, but the fruit growth was not affected much (Alegre et al. 2002). However, different organs of the plant had different sensitivity to water. Cell division and tissue expansion among different organs had independent responses to water deficit (John and Qi 2008). Cell division rate was reduced by water deficit in some plant organs, such as leaves (Aguirrezabal et al. 2006; Schuppler et al. 1998), seeds (Setter and Flannigan 2001) and roots (Sacks et al. 1997). ABA accumulation induced the expression of a cyclin-dependent kinase (CDK) activity inhibitor (Wang et al. 1998), which resulted the reducing activity of CDK (Granier et al. 2000). Hence the process of transition between the G1 and S phases of the cell cycle was blocked (Granier and Tardieu 1999). But fruits were less sensitive. According to the above explanation of root-shoot balance theory, when plants were under stress, more nutrients would be transferred to reproductive growth, giving priority to ensuring the continuity of species. Because of this phenomenon, RDI won’t have too much negative affect on crop growth (especially fruit) with saving water. In addition, these specific results were still under controversial, some researchers believed that RDI would lead to a slight reduction in fruit yield (Lu et al. 2019), but others believed that early moderate water deficit would improve yields in some specific crops (Hajian et al. 2020).

There was a clear separation between shoot growth and root growth during most periods of plant. Mild water stress could inhibited vegetative growth, reduced the growth redundancy of stem and leave, and promoted the translocation of photosynthetic assimilates to the products (Du et al. 2008). Some studies had revealed that plants with deficit irrigation at the vegetative stage increased the remobilization of carbon reserved in the vegetative tissues to the grains (Xue et al. 2006). In the normal irrigation period after RDI, the accumulated organic matter could be used to synthesize cell walls and other substances, which was related to fruit growth to compensate for the loss of photosynthetic products. When dealing with the water deficit, plant exploit mechanisms to improve crop resilience, which was regulating cell wall water content and mitigate the consequences of changes in wall hydration (Thompson and Islam 2021). When the water was excessively deficient, the stress was too heavy, or the deficient time was too long, it would cause the cell wall to lose its elasticity and fail to expand, resulting in a decline in yield. The current mainstream opinion was that RDI could be combined with dense planting, by adjusting the group structure of crops and increasing the irrigated area to achieve the same or slight increasing in yield and improving quality.

**Fig. 4:**
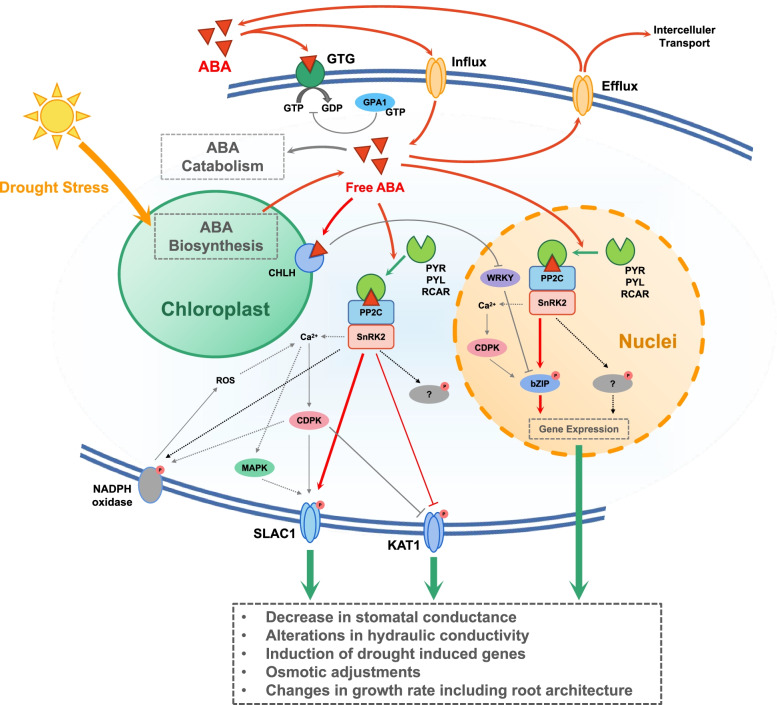
Mechanism of ABA regulation under drought stress

The ABA signaling pathway played an important role in regulating water balance and was the central to the tolerance responses of plants to drought stress (Balint and Reynolds 2013; Zhu 2016). According to the above studies, ABA was a critical factor in the mechanism of RDI action, including reducing transpiration by closing stomata and reducing shoot growth, affecting cell division and tissue expansion. In addition, ABA signaling played a key role in the accumulation of phenolic compound of fruits under RDI. Some studies have provided evidence especially in anthocyanin synthesis (Guo et al. 2021; Yang et al. 2020b). That suggest that more quality indicators are affected by ABA under RDI treatment (Fig. 4).

### RDI responses in plant

**Fig. 5:**
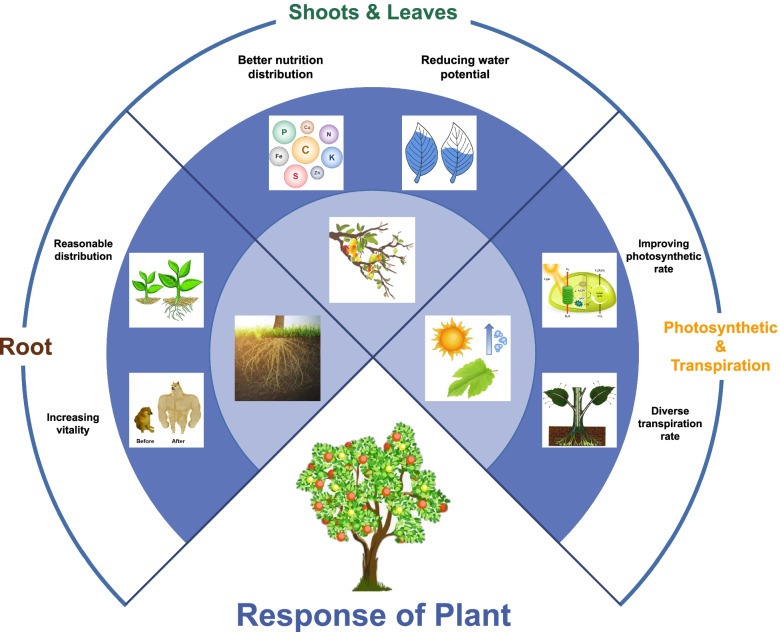
Response of plant to RDI

According to previous research progress, the response of plants to RDI had been summarized into 3 aspects: roots, shoots and leaves, photosynthesis and transpiration rates (Fig. 5).

#### Roots

The traditional concept believed that RDI treatment, especially in the seedling stage, could make crops get drought training, increase the root-shoot ratio and root activity, then promote the formation of fruit in the later stage and reduce the rate of root senescence (Li et al. 2014; Vandoorne et al. 2012; Wang et al. 2012; Yang et al. 2012). With the researches related root zone restriction in recent years, it was confirmed that root growth was not the bigger the better, plant roots in different species, different cultivation conditions had different most suitable growth distribution. Reasonable root distribution was of great significance for plant growth (Gao et al. 2021).

Root activity was an important index to evaluate the metabolic ability of plant roots, which usually directly affected the ability of absorbing soil water and nutrients (Upadhyaya and Cladwell 1993). The study of RDI to tomato had confirmed that partial RDI could increase the root activity by 48 to 59% (Yang et al. 2012). However, the root activity of some plants would not be affected by RDI. Wang et al. showed that partial root zone irrigation had no effect on rape root activity (Wang et al. 2009), which may be due to rape rooting vertically or horizontally according to soil moisture, to respond to soil water deficiency (Gan et al. 2009).

Some studies have focused on the characteristics of root growth-related gene regulatory network under water deficit, mainly including transcription factors (TFs) and regulatory small RNAs (Spollen et al. 2008). Moreover, several researchers have focused on identifying transcripts of TF gene and genome-wide networks of co-expressed genes which were differentially regulated in the primary root growth zone under water deficit stress (Opitz et al. 2014, 2016; Seeve et al. 2017). In 2019, Candace et al. identified 79 maize microRNAs that respond to specific water deficits in primary root growth zones (Seeve et al. 2019). These studies provide evidence for the mechanism of RDI affecting plant root growth.

#### Shoots and leaves

Partitioning of biomass in plants was controlled by many factors (Kobe et al. 2010; McCarthy and Enquist 2007). When fruit trees were under a moderate water stress, it would reduce the proportion of photosynthate distribution to stems, leaves and other vegetative organs, inhibit excessive vegetative growth, reduce ineffective water consumption, and promote the growth of root and reproductive organs. This tendency was beneficial to the improvement of crop quality in cash crops (Jiang et al. 2021; Lipan et al. 2021). If normal irrigation was restored before maturity, the compensation effect after rewatering could increase the distribution of photosynthates to the fruit and promote fruit growth. Some crops could even have a larger size when harvested. The nutrient in most leaves was increased with the increasing of RDI intensity, but moderate RDI treatment could decrease the content of K^+^. In all plant organs, especially in leaves, RDI significantly decreased the content of starch and increased the content of reducing sugar (Girardi et al. 2018).

The complex anatomy of plant leaves played a key role in plant growth and development, which related to the response of the water deficit (Barbour and Farquhar 2004). Detection of leaf water potential was one of the best method to measure plant water deficit, that was usually used to guide the amount of irrigation water in planting (Bastos et al. 2022). Although there were many methods of measuring the leaf water potential, it remained to be determined which method will be most accurate in the context of RDI (Atherton et al. 2012; Cheng et al. 2011; Higa et al. 2013; Sperry et al. 1996; Ullah et al. 2013; Yi et al. 2013; Zhang et al. 2012). When the leaf water potential and turgor pressure were decreasing, the normal metabolic function was disturbed, that was, water stress occurs. The mild water deficit would promote plants to reduce leaf water content and leaf water potential (Liu et al. 2006a; Perez-Pastor et al. 2014). And decreased leaf water potential trigger the reduced leaf area expansion and partial closure of stomata as a hydraulic signal (Shahnazari et al. 2007). Including the intensity of water deficit applied, plant growth stage and duration of deficit, there are many factors influence the leaf water potential during RDI treatment (Li et al. 2010; Liu et al. 2006a; Xu et al. 2011a).

The vegetative growth of plants is related to the accumulation of soluble sugars in cells, and this accumulation has an effect on nutrients, signaling and protective molecules (Bolouri-Moghaddam et al. 2010; Pagter et al. 2011). In the research of Hummel et al., water deficit led to a more positive carbon balance and activated the activities of related enzymes, and the decrease of rosette expansion rate was greater than that of photosynthesis (Hummel et al. 2010). But in the research of Vandoorne, root growth was promoted when water was deficient. This discrepancy may be due to differences in the developmental patterns of plants. Plants that involve organ enlargement after tap root initiation are more sensitive to water deficit (Vandoorne et al. 2012).

#### Photosynthesis and transpiration rates

Photosynthesis and transpiration were the basis of plant physiological process, their intensity was closely related to the environment. Photosynthesis and transpiration showed different effects in different plants under RDI treatments. Previous studies had shown that, compared with normal irrigation, moderate RDI could improve the photosynthetic rate (Romero et al. 2012). The studies of winter wheat revealed that appropriate RDI at early growth stages increased root respiration and photosynthesis (Ma et al. 2016). In the potato tuber formation and starch accumulation stages, RDI could decrease the net photosynthetic rate, stomatal conductance and transpiration rate (Li et al. 2021). Similar results had been found in grape studies (Kovalenko et al. 2021). The ratio of photosynthesis and transpiration rates also changed due to water deficit thus improving transpiration efficiency (Shabani et al. 2013). Endogenous hormone theory indicate that the water stress signal could be transmitted to leaf blades via ABA pathway, which can adjust the degree of stomatal conductance, reduce the water transpiration, and adjust the intensity of photosynthesis after receiving the signal, thereby improving the WUE and giving priority to plant reproductive growth (Speirs et al. 2013).

Rubisco is a key enzyme in photosynthesis that determines the rate of carbon assimilation and photorespiration in plants. Its response to water stress varies among plant species (Parry et al. 2002). Rubisco is present in excess in many plants, so the reduced activity and concentration of Rubisco under water stress do not significantly affect photosynthesis (Vandoorne et al. 2012). Moreover, the maintenance of Chl a/Chl b ratio was considered to be an important parameter of light phase stability under drought stress (Wang et al. 2008).

Monti et al. suggested that stomatal conductance was weakly correlated with net photosynthesis, and the change of photosynthesis was mainly caused by nonstomatal reasons (Monti et al. 2005). The research of Vandoorne et al. confirmed that NPQ (non-photochemical quenching) decreased, Qp (photochemical quenching) and φPSII (photosystem II efficiency) increased, and the efficiency of light phase increased in plant tissues under drought stress (Vandoorne et al. 2012). Similar results were found with sunflowers (Cechin et al. 2008). In *Brassica*, water-deficient irrigation at the Fruiting stage might not affect the WUE and photosynthesis of leaves, because photosynthesis at this stage mainly depended on the pod wall of the plant (Wang et al. 2009). Photosynthesis in the chloroplasts of the outer pod wall layer was the main source of assimilates for seed growth (70–100%) (Sheoran et al. 1991).

#### Sensing and signal transduction

The responses of plant to water deficit are complex and systematic. Plants sense water deficit signals, and signal transduction leads to downstream changes, which then lead to regulation at multiple levels, ultimately to phenotypic changes. Previous studies have shown that OSCA1 encoding hyperosmolarity-gated calcium channels had been identified as a primary hyperosmotic stress sensor (Zhang et al. 2022).

RDI result in hyperosmotic stress, which is thought to affect lateral tension on the membrane lipid bilayer, which can be recognized by the Ca^2+^ channels OSCA1 or OSCA1.2, resulting in Ca^2+^ spikes (Jojoa-Cruz et al. 2018). The Ca^2+^ spike has the potential to activate CPKs and CBLs-CIPKs. SnRK2s are eventually activated, resulting in ABA accumulation. ABA binds to PYLs, which then interact with and inhibit class A PP2Cs, causing SnRK2.2/3/6/7/8 to be activated (Boudsocq et al. 2004). SnRK2s that have been activated phosphorylate effector proteins such as TFs (transcription factors), SLAC1, and RbohD/F. RbohD/F produces H_2_O_2_, which triggers a Ca^2+^ signal through GHR1. This Ca^2+^ signal activates CPKs and CBLs-CIPKs, which subsequently phosphorylate effector proteins like SLAC1(Yu et al. 2014).

In addition to Ca^2+^, ABA can induce the second messengers NO (nitric oxide, which inhibits SnRK2s and PYLs) and PA (phosphatidic acid, which regulates the activity of the Rbohs protein), as well as other phospholipid signal molecules (Zhu 2016).

## Application of RDI in different crops

**Fig. 6:**
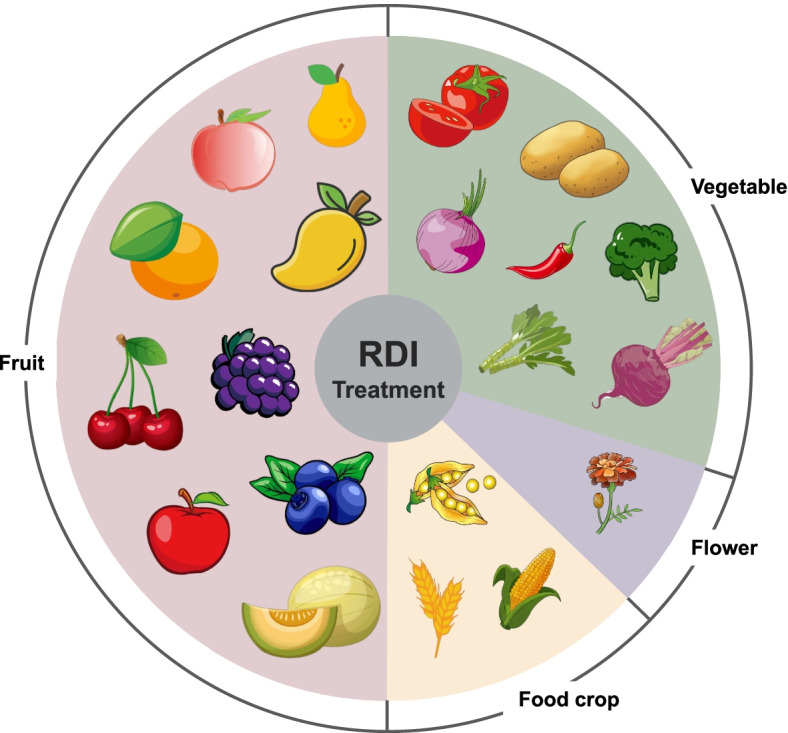
Application of RDI in different crops

At present, there are many research results about RDI, which show that it has been widely researched and applied in crops planting. These studies are roughly divided into fruit trees and vegetables for discussion (Fig. 6).

### Fruit tress

According to the research results published in recent years, RDI had been widely used in fruit trees, such as citrus, grapes, blueberries, pears, mangoes, apples, peaches, melons, and cherries. Many research results had conducted extensive and in-depth studies on the optimal deficit of RDI in different fruit trees, its specific impact mechanisms, and the effect of key indicators.

Citrus was one of the main fruits used in RDI. According to the research of Gonzalez-Dugo et al., researchers conducted RDI treatments on 2 varieties of citrus, which could save water by 50 to 55% without any impact on yield (Gonzalez-Dugo et al. 2018). It was confirmed that the application of RDI could reduce the loss caused by fruit cracking, and reduce the oversized fruits, so as to improve the commercial yield of citrus by optimizing the distribution of fruit size without affecting the sensory quality. Romero-Romero et al. proved the feasibility of using reclaimed water (RW) and RDI for medium-and-long-term irrigation of citrus (Romero-Trigueros et al. 2020). The RDI experiment conducted in the greenhouse proved that RDI was beneficial to the planting of container-grown citrus nursery trees, and would not have a significant adverse effect on the quality of the tree (Girardi et al. 2018). The RDI study of Navelina citrus trees had shown that RDI could save water by 12–27% without negative impact (Gasque et al. 2016).

Grape was widely planted in arid and semi-arid areas because of its living habits, so it was also one of the main fruits used in RDI. da Silva et al. confirmed that RDI did not affect the water potential Ψ of grape leaves, and neither RDI nor PRD affected photosynthesis and photochemical activity, and the light and dark respiration rates of leaves were not affected, so RDI did not have negative effect on carbon balance (da Silva et al. 2018). In Chardonnay (*Vitis vinifera* L.), post-harvest regulated deficit irrigation did not reduce yield, and long-term regulated deficit irrigation could affect fruit composition (Prats-Llinàs et al. 2019). The RDI experiment on grape carried out in Northwest China for many years showed that a large degree of RDI treatment could significantly increase the content of total anthocyanins and acylated anthocyanins, especially the monomer acetylated anthocyanin Malvidin. Metabonomic analysis showed that the up-regulation of the expression of some structural genes related to anthocyanin synthesis was the main reason for the change in anthocyanin content (Ju et al. 2019; Yang et al. 2020a). The studies in Spain had similar results (Pinillos et al. 2016). And under the RDI environment, the organic acid content of grapes decreased slightly, the content of glucose and fructose increased, and the expression of genes related to sugar unloading was significantly increased (Yang et al. 2020b). Recent studies had shown that adequate RDI could improve wine astringency perception by altering proanthocyanidin composition (Duan et al. 2021). The research conducted by Dayer et al. indicated that RDI affects the carbohydrate metabolism in Melbec grapevines (Dayer et al. 2013).

Similar to the results of grape research, RDI achieved satisfactory results in the planting of blueberries under the condition of moderate deficit irrigation. The yield and fruit quality (firmness, fruit size, TA, TSS and fruit weight) of deficit irrigated plants were similar to those of fully irrigated plants, and the antioxidant capacity of fully irrigated plants was higher, and severe deficit treatment would lead to greater oxidative damage, which was different from the research in grape (Lobos et al. 2018). The research on mango showed that the maximum WUE could be reached by 50–70% PRD during expansion and ripening stage (Dos Santos et al. 2016). In research of apple trees, the best period of RDI treatment was from flowering to fruit setting, when the WUE was the highest, and the treatment at this time had obvious positive effect on apple quality, the effect of 55–70% deficit treatment was the best (Zhong et al. 2019). The research of RDI on apple trees results that neither RDI treatment significantly affected shoot growth, fruit growth, photosynthesis, and stomatal conductance (Hooijdonk et al. 2004). For peach trees research, it was found that the degree and time of RDI had significant effects on the antioxidant compounds in fruit tissues, and the RDI treatment group had higher concentrations of bioactive compounds (Falagan et al. 2016). In addition, RDI treatment significantly reduced the pruning weight of peach trees, but there was no difference in reproductive growth and yield, which was similar to the results in other crops (Manuel Miras-Avalos et al. 2017). Pear tree was also one of the target crops treated with RDI. Wu et al. compared the effects of PRD and traditional RDI treatments on pear trees and found that under extreme drought conditions, the leaves gas exchange, vegetative growth and fruit growth of pear were mainly affected by irrigation amount, but not by different irrigation methods. That was to say, the effects of the two deficit irrigation methods were similar in pear trees (Wu et al. 2020).

For fruit trees, the research content of RDI focuses on how to improve the quality of fruit through RDI. Therefore, researchers paid attention to fruit quality indicators under RDI treatments, such as Soluble solids, polyphenols, anthocyanins, and bioactive compounds. For fruit trees, better quality usually means better economics.

### Vegetables

Not only fruit trees, but also vegetables were a major application field of RDI. Li et al. applied 7 treatments according to the growth and development stage of sugar beet (canopy development, root development and sugar accumulation) and the degree of water deficit (30, 50 and 70% of field capacity, FC). The yield, quality, sugar yield, irrigation WUE and main agronomic physiological characters of sugar beet were evaluated. During canopy development, moderate water deficit (50% of FC) increased sugar production by 27% compared with the control (70% of FC during the whole growth period). Severe water deficit (30% of FC) increased sugar production by 45% during storage root development and 55% during sugar accumulation. RDI during canopy development and storage root development inhibited leaf growth, but did not affect yield (Li et al. 2019).

The joint experiment of RDI and selenium application on cauliflower showed moderate RDI was the most effective strategy to maintain the yield of cauliflower, improved the antioxidant capacity and phenolic compound content. Foliar spraying selenium increased the content of total polyphenols under drought stress. Heavy RDI (50%) prolonged the shelf life of cauliflower (Hachmann et al. 2019). More specific studies on economic benefits confirmed that moderate or severe RDI in childhood did not significantly reduce head yield compared with fully irrigated plants (4.4 kg/m^2^)。As a result, a similar gross income was obtained (16,859 Euros/ha), but a large amount of water was saved (up to 24.3%) and integrated WUE was improved (up to 34.2 kg/m^3^) (Abdelkhalik et al. 2019a).

Onions were a common food in many countries in arid and semi-arid regions. The researchers previously used 5 irrigation treatments, including application 100% irrigation water requirement (IWR) throughout the growing periods, and 75% or 50% IWR during vegetative growth, and bulb maturity. The deficit irrigation strategy tested reduced the yield more or less, therefore, if water was available at any time, adequate irrigation is recommended. When application 50% IWR with RDI during bulb maturity, the water saving effect was significant (22%), the yield decreased slightly (9%), and the irrigation water use efficiency (IWUE) increased by 20% compared with adequate irrigation group. It could be recommended in the case of severe water shortage. At the bulb maturity, RDI achieved a satisfactory yield with 75% IWR, resulting in a slightly decrease in yield (4%) and a great increase in IWUE (9%). The experiment confirmed that RDI was a desirable strategy for onion planting under mild water shortage in the Mediterranean region (Abdelkhalik et al. 2019b).

In order to explore the application conditions of RDI in pepper planting, Yang and others carried out RDI experiments on pepper with different degrees of deficit in different stages. Their results showed that the RDI strategy reduced the actual crop evapotranspiration by 2–27%, and the yield was the highest under full irrigation. When water deficit occurs in the middle stage, the yield reduction was the largest (13–20%). The reduction of the yield in 50% water deficit group was more than 25% group. When there was water deficit in the later stage, the productivity and WUE were the highest. RDI might improve pepper quality by increasing the content of soluble solids in fresh pepper. The guiding conclusion for production was that in order to obtain the maximum yield, adequate irrigation was recommended first, if the economic benefits and WUE of the region are taken into account, the water deficit in the later stage is recommended to be 25–50% (Yang et al. 2018).

The research focus of vegetables under RDI treatments was different from fruit trees. In addition to the basic quality indicators, people paid more attention to the storage period of vegetables after RDI treatment, WUE, etc. But as with fruit tree research, it is how much the economics can be improved through RDI that matters.

## Challenge and prospects

### Deficiency of current research

The function, illumination, plant response, and application of RDI in different crops are summarized above. Although many studies have confirmed that RDI is an effective way to improve crop quality and economy, there are still many deficiencies and gaps in current research.

There is still currently a lack of consensus on what is RDI and which irrigation methods should be included in the scope of RDI. This review summarizes this, and roughly divides it into three types: namely, Stage-based deficit irrigation, Partial root-zone irrigation/Partial root drying (PRD), and Subsurface irrigation. Other irrigation methods based on precise water control measures may be derived in the future, but should also be considered as belonging to RDI.

Crop species suitable for RDI are incomplete. In addition to the crops listed in this review, more crops that are suitable for RDI may be studied in the future; or as development of RDI methods, crops that are not suitable for RDI now may also be suitable.

The specific mechanism of RDI is still unclear. Although RDI has been used in a variety of crops, the existing research has paid more attention to its effect, but its mechanism of action is still incomplete. The research of that would involve a lot of knowledge related to cell biology and molecular biology. With the in-depth study of plant mechanism, the mechanism of RDI would become more unequivocal.

Lack of simple, effective application procedures. Application is the purpose of technology, so more researchers are needed to summarize and simplify RDI methods and effects, which can provide a simple and feasible procedure of RDI treatment for specific crops in specific environments. This procedure can be used in agricultural production to obtain the expected advantages of RDI.

### Difficulties in large-scale promotion

RDI is suitable for arid and semi-arid regions, especially those with urgent water needs, so people in other regions did not pay much attention to this method. However, based on the previous researches, those areas with sufficient water may also improve the quality of crops and improve their economy through RDI. However, under RDI conditions, the vegetative growth of plants is weakened, which is contrary to the traditional planting purpose of lush foliage, so it is difficult to be accepted by people.

Cost is also a big problem for promotion. Since RDI treatment is based on precise water control, drip irrigation and water measurement equipment are required. The construction and maintenance of these equipment requires a certain cost, which brings difficulties to large-scale promotion. Therefore, there is an urgent need for specific RDI application procedures for specific crops, which can model the construction of RDI equipment and is conducive to promotion.

## Conclusion

Water was one of the most important elements for the survival of all terrestrial plants, and in the vast arid and semi-arid areas of the world, the lack of precipitation and water resources year-round or seasonally seriously restricted the economic development and the improvement of people’s living. In order to solve the worldwide shortage of water resources, a variety of water-saving strategy had been implemented in agricultural production. With the exploration of the theory of plant water demand and the in-depth study of modern horticulture, RDI as a new water-saving and quality-improving irrigation measure was being accepted and popularized by the majority of horticultural crop growers.

This review summarized the basic situation, the specific function in production, the theory of action of RDI and the response of plants to it, as well as the application of RDI in various horticultural crops. RDI played a similar but different role in different horticultural crops. Before the maturity, especially in the early stage of plant growth, light RDI treatment could play a well role in water saving and quality improvement. Moreover, RDI could induce plants to make appropriate physiological adjustments and enhance the resistance to stress of plants. However, in practice, RDI was also found that there are still many problems to be solved, and the basic theory of quality enhancement needs to be completed. At present, there was still a lack of specific measures and regulations for RDI under different crops, different soil types and different environmental conditions, which still need to be improved and explored by researchers.

## Data Availability

Yes.
